# Strains and Stressors: An Analysis of Touchscreen Learning in Genetically Diverse Mouse Strains

**DOI:** 10.1371/journal.pone.0087745

**Published:** 2014-02-19

**Authors:** Carolyn Graybeal, Munisa Bachu, Khyobeni Mozhui, Lisa M. Saksida, Timothy J. Bussey, Erica Sagalyn, Robert W. Williams, Andrew Holmes

**Affiliations:** 1 Laboratory of Behavioral and Genomic Neuroscience, National Institute on Alcoholism and Alcohol Abuse, National Institutes of Health, Bethesda, Maryland, United States of America; 2 Departments of Preventive Medicine, and Department of Anatomy and Neurobiology, University of Tennessee Health Science Center, Memphis, Tennessee, United States of America; 3 Department of Experimental Psychology, University of Cambridge, Cambridge, Medical Research Council and Wellcome Trust Behavioral and Clinical Neuroscience Institute, Cambridge, United Kingdom; University of Chicago, United States of America

## Abstract

Touchscreen-based systems are growing in popularity as a tractable, translational approach for studying learning and cognition in rodents. However, while mouse strains are well known to differ in learning across various settings, performance variation between strains in touchscreen learning has not been well described. The selection of appropriate genetic strains and backgrounds is critical to the design of touchscreen-based studies and provides a basis for elucidating genetic factors moderating behavior. Here we provide a quantitative foundation for visual discrimination and reversal learning using touchscreen assays across a total of 35 genotypes. We found significant differences in operant performance and learning, including faster reversal learning in DBA/2J compared to C57BL/6J mice. We then assessed DBA/2J and C57BL/6J for differential sensitivity to an environmental insult by testing for alterations in reversal learning following exposure to repeated swim stress. Stress facilitated reversal learning (selectively during the late stage of reversal) in C57BL/6J, but did not affect learning in DBA/2J. To dissect genetic factors underlying these differences, we phenotyped a family of 27 BXD strains generated by crossing C57BL/6J and DBA/2J. There was marked variation in discrimination, reversal and extinction learning across the BXD strains, suggesting this task may be useful for identifying underlying genetic differences. Moreover, different measures of touchscreen learning were only modestly correlated in the BXD strains, indicating that these processes are comparatively independent at both genetic and phenotypic levels. Finally, we examined the behavioral structure of learning via principal component analysis of the current data, plus an archival dataset, totaling 765 mice. This revealed 5 independent factors suggestive of “reversal learning,” “motivation-related late reversal learning,” “discrimination learning,” “speed to respond,” and “motivation during discrimination.” Together, these findings provide a valuable reference to inform the choice of strains and genetic backgrounds in future studies using touchscreen-based tasks.

## Introduction

Touchscreen-based systems for testing learning and cognition in rodents are growing in popularity. Because closely matched methods can be used to evaluate cognition in patients with neuropsychiatric disorders, touchscreen-based methods in mice may have translational relevance that in turn may foster improved bidirectional translational studies of cognitive impairment and cognitive enhancing therapeutics [1]–[2].

Previous studies employing touchscreen-based assays in mice have begun to elucidate the neural systems underlying performance in certain cognitive tasks [3]–[4], and have demonstrated effects of gene mutations [5]–[10], pharmacological treatments [5], [8], [11]–[12] and ‘insults’ from drug or stress exposure [13]–[14] on performance. Several recent studies have also described optimal procedural parameters used in mice for detecting experimental effects on cognition, including attention, paired-associates, extinction, discrimination, and reversal learning [15]–[17]. Together, these prior findings provide preliminary support for the reliability and validity of the touchscreen platform as an approach to study the neural mechanisms of cognition and cognitive disorders.

The selection of appropriate strains, or genetic background in the case of genetically modified mouse lines, is of critical importance for experiments using touchscreens, because strains are well known to differ in learning in ‘classical’ assays. For example, mouse strains have been shown to differ from one another in maze-based measures of learning [18]–[21], as well as operant-based measures of attention [22]–[23], impulsivity [22],[24]–[26] and instrumental learning [15], [27]. Comparing the effects of experimental manipulations across strains can be difficult when strains exhibit abnormal baseline performance levels [28]. The demonstration of reliable strain differences in touchscreen learning could also provide a basis to identify neural and genetic factors underlying learning in this setting [29]. However, potential performance variation between different mouse strains in these assays has not been well described.

In the current study we have tested a family of diverse mouse strains for differences in behavior using the touchscreen platform. We focus on two forms of learning that have been commonly examined with this system: pairwise visual discrimination and reversal learning. We began with an analysis of seven of the most widely-used inbred mouse strains in behavioral research. We detected significant variation among these strains, including learning differences between DBA/2J and C57BL/6J. We conducted additional experiments on these two strains as they are among the most frequently used in behavioral neuroscience, including in our previous touchscreen studies [15], and are the parental strains of BXD recombinant inbred (RI) strains tested in the current study [30]. We examined whether basal differences in learning between these strains extended to differential sensitivity to stress, based on the finding that stress facilitates reversal learning in C57BL/6J [13], as well as evidence that DBA/2J and C57BL/6J differ in stress responses on other behavioral (i.e., anxiety-related) measures [31]. Next, as an initial step towards the identification of genetic factors underlying learning differences in these strains, we phenotyped a panel of BXD strains. Each of these BXD strains comprises a unique combination of just over five million segregating sequence variants inherited from the two parental strains [30]. Finally, we examined the behavioral structure of discrimination and reversal learning via principal component analysis of a large dataset (current plus archival) of ∼800 mice.

## Materials and Methods

### Subjects

Male 129S1/SvImJ, A/J, BALB/cJ, BALB/cByJ, C57BL/6J, DBA/2J, and FVB/NJ inbred mice were obtained from the Jackson Laboratory (Bar Harbor, ME, USA). BXD mice were bred at the University of Tennessee Health Science Center and shipped to NIAAA for phenotyping. We used the following 27 BXD strains: BXD9, 12, 13, 27, 29, 32, 34, 39, 40, 43, 48, 55, 60, 61, 62, 69, 73, 74, 87, 95, 96, 100, 102, 122, 124, 138, 149. BXD96 is now known as BXD48a (JAX stock number 007139) and is ∼94% identical by descent with the original BXD48 (JAX stock number 008097) that we also studied. All of the BXD strains that we have studied are available from the Jackson Laboratory, with the exceptions of the new BXD strains: BXD122, BXD124, BXD138, and BXD149. This is the first study to use these latter strains.

Mice were obtained at 7–8 weeks of age and housed by strain in a temperature (72±5°F) and humidity (45±15%) controlled *vivarium* under a 12-hour light/dark cycle (lights on at 0700 h, lights off at 1900 h), to which they acclimated to for at least 1 week prior to testing. Testing occurred between 0900–1500 h. All experimental procedures were approved by the NIAAA Animal Care and Use Committee and followed the NIH guidelines outlined in ‘Using Animals in Intramural Research’ and the local Animal Care and Use Committees, and all efforts were made to minimize suffering.

#### Inbred strain survey

129S1/SvImJ, A/J, BALB/cJ, BALB/cByJ, C57BL/6J, DBA/2J, and FVB/NJ mice were compared for visual discrimination and reversal learning. These strains were chosen on the basis of their widespread use in neuroscience, our own previous strain analyzes [29], [31]–[33], and their inclusion as ‘Group A’ strains in the Mouse Phenome Project - an effort to phenotypically characterize hundreds of mouse strains in order to inform the selection of appropriate strains for experimentation [34] (www.jax.org/phenome).

### Apparatus

The apparatus and testing procedures were as previously described (e.g., [6], [9], [13], [35]). Testing was conducted in an operant chamber (21.6×17.8×12.7 cm (model # ENV-307W, Med Associates, St. Albans, VT) that was housed within a sound- and light-attenuating box (Med Associates, St. Albans, VT, USA), with the floor covered with solid Plexiglas to aid ambulation. At one end of the chamber, 14 mg dustless pellets were dispensed by a pellet dispenser (#F05684, BioServ, Frenchtown, NJ, USA) into a magazine. At the other end of the chamber, a touch-sensitive screen (Light Industrial Metal Cased TFT LCD Monitor, Craft Data Limited, Chesham, U.K.), there was a house-light, and a tone generator. The touchscreen had 2×5 cm windows separated by 0.5 cm located at 6.5 cm above the floor. Visual stimuli, visible through the windows (1 stimulus/window), were presented on the screen and controlled by custom-made software (‘MouseCat’, L.M. Saksida). Touchscreen nosepokes at the stimuli were recorded by the software.

### Pre-training

Mice were first slowly reduced and then maintained at 80–85% free-feeding body weight. Prior to testing, mice were acclimated to the 14 mg pellet food reward by provision of ∼10 pellets per mouse in the homecage for 1–3 days. Mice were then acclimated to the operant chamber and to eating out of the pellet magazine by being placed in the chamber for 30 minutes with pellets available in the magazine. Mice that ate 10 pellets within 30 minutes were moved onto autoshaping which consisted of variously shaped stimuli being presented in the touchscreen windows (1 per window) for 10 seconds (inter-trial interval (ITI) 15 sec). The disappearance of the stimuli coincided with delivery of a single pellet food reward, concomitant with presentation of 2 ‘conditioned reinforcer stimuli’ (2-sec 65-dB auditory tone and illumination of pellet magazine) that served to support instrumental learning. Pellet retrievals from the magazine were detected as a head entry and, at this stage of pre-training, initiated the next trial. To encourage screen approaches and touches at this stage, nosepokes at the touchscreen delivered 3 pellets into the magazine.

Mice retrieving 30 pellets within 30 minutes were moved onto the ‘touch’ phase of touchscreen pre-training. Here, mice obtained rewards by responding to stimuli of different shapes that appeared in one of the two windows (spatially pseudorandomized) that remained on the screen until a response was made (‘respond’ phase). Mice retrieving 30 pellets within 30 minutes were then moved on to the ‘punish’ phase, in which they were required to initiate each new trial with a head entry into the pellet magazine. In addition, responses at a blank window during stimulus presentation now produced a 15 second timeout (signaled by extinction of the house light) to discourage indiscriminate responses. Errors were followed by correction trials in which the same stimulus and left/right position were presented until a correct response was made. Mice making ≥75% (excluding correction trials) of their responses at a stimulus-containing window over a 30-trial session were moved onto discrimination. Mice that did not reach criterion for each phase within 20 sessions were not tested for discrimination.

### Discrimination

Two novel, approximately equiluminescent stimuli, were presented in a spatially pseudorandomized manner over 30-trial sessions (15 second ITI). One stimulus was designated as the CS+ and the other as the CS− (pseudorandomly across mice). Responses at the CS+ resulted in reward; while responses at the CS− resulted in a 15 second timeout (signaled by extinction of the house light) and were followed by a correction trial. Stimuli remained on screen until a response was made. Designation of the correct and incorrect stimulus was counterbalanced across groups. Mice were trained until ≥85% of responses (not including correction trials) were at the correct stimulus on each of two consecutive sessions. Mice that did not reach criterion for each phase within 60 sessions were not tested for reversal.

### Reversal

Reversal testing began on the session after discrimination criterion was attained. Here, the designation of the CS+ and CS− was reversed. Mice were trained until ≥85% of responses (not including correction trials) were at the correct stimulus on each of two consecutive sessions. Testing was terminated if mice did not attain criterion within 60 sessions.

### Statistical analysis

The dependent variables were the number of sessions to attain each sub-stage of pre-training, and the number of sessions, errors and correction errors to reach discrimination and reversal criterion. Average stimulus-reaction time and reward latency during discrimination and reversal were measured. Errors and correction errors during the early and late stages of reversal were calculated, as errors/correction committed on sessions where percent correct performance was below chance ( = early) or equal to/greater than chance (late), as previously described [4], [13]. Measures were compared across strains using analysis of variance (ANOVA), followed by Fisher's Least Significant Difference *post hoc* tests, using C57BL/6J as the reference to which other strains were compared.

#### Effects of stress on reversal learning in C57BL/6J and DBA/2J mice

We assessed whether baseline differences in reversal learning between the DBA/2J and C57BL/6J strains extend to differential responsivity to an environmental manipulation in the form of stress. Mice were trained to discrimination criterion (as described above) and then subjected to forced swim stress once on each of the following three consecutive days (between 1400–1600 h). The mouse was placed in a 20-cm diameter Plexiglas cylinder half-filled with 24±1°C water for 10 minutes, and then returned to the homecage [13]. Non-stressed controls remained in their homecage. Reversal testing began the day after the final swim. Touchscreen testing was the same as described above, with the exception that the number of trials per session was increased from 30 to 60 for consistency with our prior study design [13]. Data were compared across reversal learning stages using repeated measures ANOVA, and between strains and stress groups using a 2-factor ANOVA and Fisher's Least Significant Difference *post hoc* tests.

#### BXD analysis

To explore the genetic basis of differences in discrimination and reversal learning, we tested a panel of 27 BXD strains for discrimination and reversal using the same procedures as above. For comparison, the DBA/2J and C57BL/6J parental strains were tested along with the BXD lines. Given evidence of phenotypic differences between C57BL/6J and C57BL/6NJ substrains [36]–[37], C57BL/6NJ mice were also included. In addition, given the previous finding that DBA/2J are slower to extinguish a non-rewarded simple single-stimulus-reward touchscreen response than C57BL/6J [15], DBA/2J were also tested for extinction of learned responses, beginning the session after reversal criterion was attained. The procedure for extinction was the same as reversal except that touchscreen responses at either stimulus had no programmed consequences. Mice were trained on 30-trial daily sessions to a criterion of making <25% responses over two consecutive sessions.

Strains were compared using ANOVA, as for the inbred strain survey above. Behavioral measures were also correlated to estimate of genetic associations between the various forms of learning and performance [38].

#### Principal components analysis

To complement the BXD analysis, we also conducted a multivariate analysis of discrimination and reversal using principal component analysis (PCA) on a total of 765 mice, as previously applied to other mouse behaviors [39]–[41]. The data comprised all subjects used in the current study, as well as previously tested C57BL/6J mice or mutants on a C57BL/6J genetic background. All mice were tested in the current laboratory, using the procedures described above, over a period of approximately seven years.

The structure of the following 15 behavioral measures was defined using PCA: sessions to pre-training, discrimination and reversal criteria, errors and correction errors to discrimination and reversal criteria, errors and correction errors to early (below-chance) reversal criteria, errors and correction errors to late (above-chance) reversal criteria, average stimulus-reaction times, and reward-retrieval latency during discrimination and reversal. PCA was conducted using the R package FactoMineR [42] (http://www.r-project.org/). Any missing observations (1,719 of 11,475) were replaced by variable means and an orthogonal linear transformation was applied to define the principal components. Only the components with eigenvalues greater than the mean (>1) were retained for analysis.

## Results and Discussion

### Inbred strains differ in learning

#### Pre-training performance

**Figure 1:**
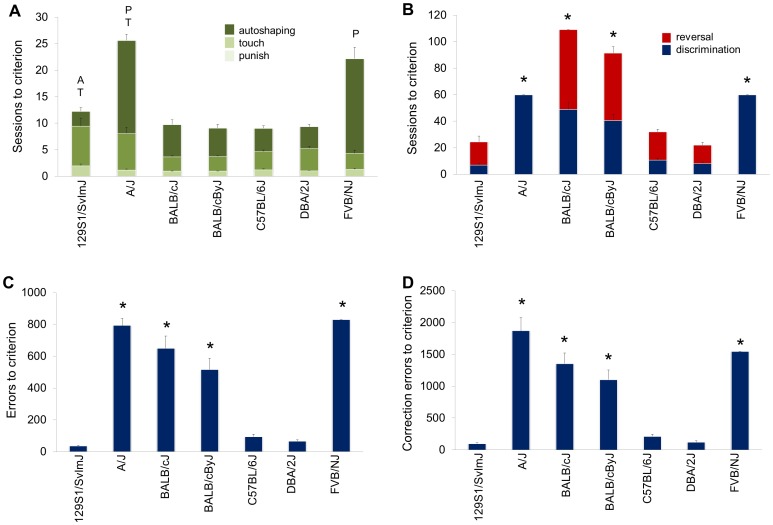
Inbred strain differences in pre-training performance, and in discrimination and reversal learning. (**A**) Sessions to autoshaping, touch and punish criterion. (**B**) Sessions to discrimination and reversal criterion. Errors (**C**) and correction errors (**D**) to discrimination criterion. ^A^ *P*<.05 autoshaping session relative to C57BL/6J, ^T^ *P*<.05 touch sessions relative to C57BL/6J, ^p^ *P*<.05 punish session relative to C57BL/6J, *P<.05 discrimination, reversal, errors or correction errors relative to C57BL/6J. n = 9–27 per strain. Data are Means ± SEM.

There were significant strain differences in the number of sessions to attain criterion for the autoshaping (F6,99 = 4.15, *P*<.01), touch (F6,98 = 6.98, *P*<.01), and punish (F6,96 = 44.49, *P*<.01) phases of pre-training (**Figure 1A**). Of 15 BALB/cByJ, 14 A/J and 9 FVB/NJ mice that were tested, 8%, 64% and 86% (respectively) failed to complete pre-training within 20 sessions/stage, primarily due to a failure to complete the punish phase. The poor pre-training (and discrimination, see below) in the FVB/NJ strain is most likely due to impaired vision caused by inheritance of the *Pde6^rd1^* retinal degeneration mutation [43]–[45]. Poor vision in the A/J strain has also been reported in other learning settings [44], [46] and could account for the pre-training and discrimination (see below) difficulties found in the current study.

#### Discrimination learning

Strains differed significantly in sessions (F6,79 = 40.78, *P*<.01), errors (F6,79 = 36.06, *P*<.01) and correction errors (F6,79 = 37.61, *P*<.01) to discrimination criterion, as well as average stimulus-reaction time (F6,79 = 4.63, *P*<.01). The number of sessions, errors and corrections errors was significantly higher (*P*<.05) in A/J, BALB/cJ, BALB/cByJ, and FVB/NJ mice than C57BL/6J mice but significantly lower in 129S1/SvImJ mice for sessions and errors than C57BL/6J mice (**Figure 1B–D**). Reward-retrieval latency was slower in 129S1/SvImJ mice than C57BL/6J (Figure S1A–B). All the A/J and FVB/NJ mice tested on discrimination failed to reach criterion within 60 sessions, again likely because of visual problems. Of the 12 BALB/cJ and 11 BALB/cByJ tested, 58% and 38% (respectively) failed to reach discrimination criterion within 60 sessions. Both strains also showed significantly faster stimulus-reaction times relative to C57BL/6J (Figure S1A), an observation that could relate to increased impulsivity. However, BALB/cJ mice are similar to C57BL/6J mice on more direct measures of impulsivity [25]–[26], and the exact nature of the discrimination learning impairments remains unclear.

#### Reversal learning

Of the five strains that were tested, there were significant differences in sessions (F4,61 = 34.85, *P*<.01), errors (F4,61 = 77.23, *P*<.01) and correction errors (F4,61 = 62.50, *P*<.01) to reversal criterion, and also in average stimulus-reaction time (F4,61 = 4.38, *P*<.01) and reward-retrieval latency (F4,61 = 3.65, *P*<.01). All BALB/cJ and six of the seven BALB/cByJ mice tested failed to reach criterion within 60 sessions. The number of sessions, errors and corrections errors was significantly higher in BALB/cJ and BALB/cByJ than in C57BL/6J, and were significantly lower in 129S1/SvImJ and DBA/2J than C57BL/6J (Figure 1B, 2A–B). Stimulus-reaction times and reward-retrieval latencies were slower in 129S1/SvImJ than C57BL/6J (Figure S1C–D).

**Figure 2:**
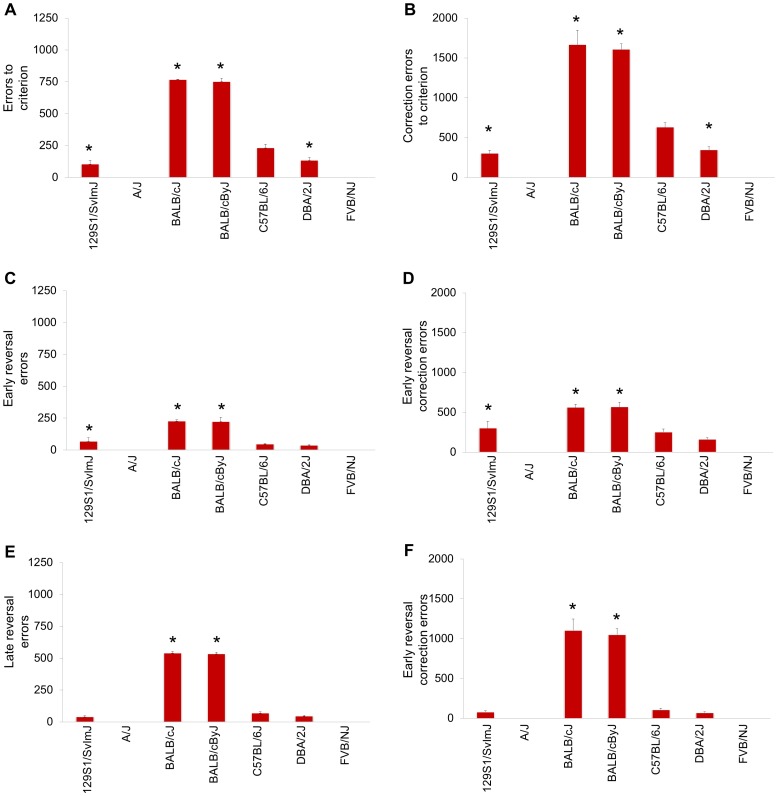
Inbred strain differences in reversal learning. Errors (**A**) and correction errors (**B**) to reversal criterion. Errors (**C**) and correction errors (**D**) made during early reversal. Errors (**E**) and correction errors (**F**) made during late reversal. *P<0.5 relative to C57BL/6J. n = 2–27 per strain. Data are Means ± SEM.

For the early reversal phase alone, there were significant strain differences in errors (F4,61 = 18.38, *P*<.01) and correction errors (F4,61 = 13.45, *P*<.01). BALB/cByJ and BALB/cJ mice made significantly more errors and BALB/cByJ made significantly more correction errors than C57BL/6J (**Figure 2C–D**). For the late reversal, there were significant strain differences in errors (F4,61 = 32.22, *P*<.01) and correction errors (F4,61 = 44.38, *P*<.01). BALB/cByJ and BALB/cJ made significantly more errors and corrections errors than C57BL/6J mice (**Figure 2E–F**).

In sum, this survey of seven inbred strains revealed major quantitative differences in touchscreen learning and performance. In contrast to the poor performance of the A/J, FVB/NJ, BALB/cJ, and BALB/cByJ strains, C57BL/6J, DBA/2J and 129S1/SvImJ successfully completed testing at every stage. Learning in 129S1/SvImJ was excellent, a finding which concurs with the good learning of this strain in tests including Pavlovian fear conditioning and assays of visual discrimination [46]–[48], but contrasts with deficient performance in tasks such as the Barnes maze, which may be compromised by low exploration phenotype of this strain [32]. Another key observation was the faster discrimination and reversal learning in DBA/2J, relative to C57BL/6J. This replicates a preliminary strain comparison of discrimination and reversal learning involving these strains [35], and extends reports of strain differences using other touchscreen tasks [15].

While DBA/2J are faster learners in these touchscreen-based tasks, this strain typically performs more poorly than C57BL/6J on spatial and contextual tasks that rely heavily on hippocampal function (e.g., [49]–[54]), whereas on non-hippocampal tasks, including visual and olfactory discrimination learning, DBA/2J perform better than C57BL/6J [48], [55]. This indicates a highly task-specific pattern of strain differences and implies that some brain systems (e.g., hippocampal) may be relatively impaired in DBA/2J, while others important for discrimination and reversal learning (e.g., prefrontal, dorsal striatal) [4], [13] may be ‘hyperfunctional’ relative to C57BL/6J. Although neural substrates of such differences remain poorly understood, DBA/2J have stronger midbrain dopaminergic immunoreactivity and innervation of prefrontal cortex [56]–[59]. Given the role of dopamine and these brain regions in learning, this could be one avenue to explore as a mechanisms contributing to the excellent touchscreen discrimination and reversal learning of DBA/2J.

### Differential effects of stress on reversal learning in C57BL/6J and DBA/2J mice

Our next step was to further explore the difference in reversal learning between C57BL/6J and DBA/2J mice, by assessing whether these strains also varied in their responses to stress effects on reversal learning. As noted in the Introduction, we focused on these two strains because of their frequent use in behavioral neuroscience and for the fact they are the parental strains of BXDs which tested in the current study. We have previously shown that three daily exposures to forced swim stress facilitates reversal learning in C57BL/6J mice, as manifest in fewer errors and correction errors made to reach criterion levels of performance relative to non-stressed controls [13].

#### Pre-stress discrimination learning

On the discrimination task, prior to stress exposure, DBA/2J mice showed non-significant trends for fewer sessions (DBA/2J = 9.38±0.81, C57BL/6J = 11.96±1.16, *t*(43) = 1.77, *P* = .08), errors (DBA/2J = 77.76±8.98, C57BL/6J = 106.38±14.06, *t*(43) = 1.66, *P* = .10), and correction errors (DBA/2J = 153.52±18.80 correction errors, C57BL/6J = 238.96±42.18, *t*(43) = 1.75, *P* = .09) to discrimination criterion. On average, DBA/2J mice had significantly longer stimulus-reactions times (DBA/2J = 9.35±0.70, C57BL/6J = 6.50±0.79, *t*(43) = −2.65, *P*<.05), and shorter reward-retrieval latencies (DBA/2J = 1.65±0.06, C57BL/6J = 2.22±0.13, *t*(43) = 3.89, *P*<.01) than C57BL/6J mice during discrimination learning. While not as robust as the differences observed in our earlier experiments, these data are generally consistent with the faster touchscreen discrimination learning in DBA/2J mice, as compared to C57BL/6J mice, seen in the broader strain survey discussed above, as well as earlier studies [35].

#### Post-stress reversal learning

**Figure 3:**
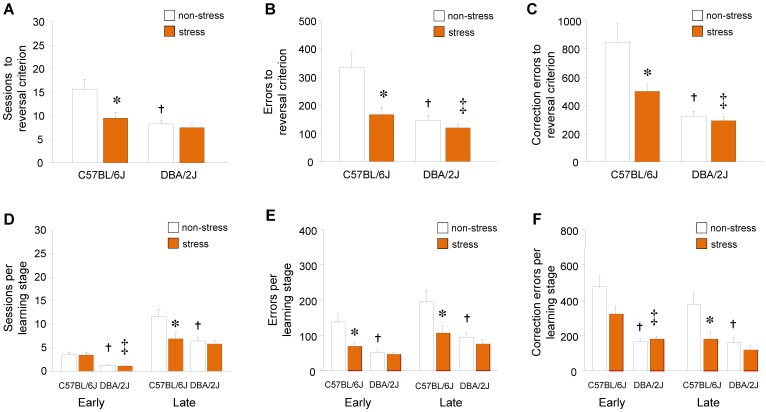
Stress effects on reversal learning in DBA/2J and C57BL/6J mice. (**A**) Sessions to reversal learning criterion. Errors (**B**) and correction errors (**C**) to reversal criterion. Sessions to early and late stage reversal (**D**). Errors (**E**) and correction errors (**F**) made during early and late stage reversal. *P<.05 stressed vs. non-stressed control C57BL/6J, †*P*<.05 non-stressed DBA/2J vs. non-stressed C57BL/6J, ‡*P*<.05 stressed DBA/2J vs. stressed C57BL/6J. n = 10–13 per stress group, per strain. Data are Means ±SEM.

On reversal testing, following exposure to 3 days of forced swim stress, there was a significant strain×stress interaction for errors (F1,41 = 4.17, *P* = .05), and a trend for a strain×stress interaction for sessions (F1,41 = 3.52, *P* = .07) and for correction errors (F1,41 = 3.89, *P* = .06), to reversal criterion. Although significant interaction terms were not found for all measures, we justified conducting planned post hoc comparisons to examine the effects of stress in each strain based on the hypothesis that the strains would differentially respond to stress exposure. This analysis revealed significantly fewer reversal sessions, errors and correction errors in stressed C57BL/6J than in non-stressed C57BL/6J control, but no effects of stress on any of measure in DBA/2J (**Figure 3A–C**). There were, however, fewer errors and correction errors in stressed DBA/2J than in stressed C57BL/6J. Lastly, further reflecting strain differences in basal reversal performance, there were significantly fewer sessions, errors, and correction errors in non-stressed DBA/2J than non-stressed C57BL/6J. There was also a significant main effect of strain (F1,41 = 24.43, *P*<.01) for average stimulus-reaction times due to slower times in DBA/2J than C57BL/6J mice (Figure S2A).

These data replicate the previously reported stress-facilitation of reversal learning in the C57BL/6J [13], and show that this effect is absent in the DBA/2J strain.

#### Post-stress early reversal

In our prior report in C57BL/6J, stress effects were largely limited to the late stage of reversal learning, when performance was above chance levels. Moreover, performance differed during the early and late phases of reversal for all measures in C57BL/6J (sessions: C57BL/6J: F1,22 = 41.58, *P*<.01, errors: F1,22 = 11.42, *P*<.01, correction errors: F1,22 = 12.84, *P*<.01) and for most variables in DBA/2J (sessions: F1,18 = 101.51, *P*<.01, errors: F1,18 = 13.38, *P*<.01, correction errors: *P*>.05). We therefore parsed the current data into early and late reversal stages. During early reversal, there was a significant stress×strain interaction for errors (F1,41 = 4.92, *P*<.05), and a main strain effect for sessions (F1,41 = 21.15, *P*<.01), correction errors (F1,41 = 26.11, *P*<.01), stimulus-reaction time (F1,41 = 18.22, *P*<.01), and reward-retrieval latency (F1,41 = 5.17, *P*<.05). Planned post hoc comparisons were again conducted and indicated fewer errors in stressed C57BL/6J as compared to non-stressed C57BL/6J, as well as fewer sessions and correction errors in stressed DBA/2J mice, as compared to stressed C57BL/6J mice (**Figure 3D–F**). There were also fewer sessions, errors and correction errors, slower stimulus-reaction times and faster reward-retrieval latencies, in non-stressed DBA/2J mice than non-stressed C57BL/6J counterparts (**Figure 3D–F**, Figure S2C–D).

These data confirm the absence of robust effects of stress during the early reversal stage, either in C57BL/6J (as previous observed [13]), or DBA/2J.

#### Post-stress late reversal

During late reversal, there was a significant effect of strain and stress on sessions (strain: F1,41 = 23.96, *P*<.01; stress: F1,41 = 4.90, *P*<.05), errors (strain: F1,41 = 7.51, *P*<.01; stress: F1,41 = 6.40, *P*<.05) and correction errors (strain: F1,41 = 8.24, *P*<.01, stress: F1,41 = 6.85, *P*<.05), and a significant effect of strain for stimulus-reaction times (F1,41 = 23.96, *P*<.01). Planned post hoc comparisons showed significantly fewer sessions, errors and correction errors in stressed than non-stressed C57BL/6J groups, but no differences between DBA/2J groups (**Figure 3E–F**). There were fewer sessions, errors and correction errors (**Figure 3E–F**), and slower stimulus-reaction times (Figure S2C), in non-stressed DBA/2J mice than non-stressed C57BL/6J, reflecting the basal strain difference in performance. Thus, these data confirm the facilitatory effect of stress during late reversal in C57BL/6J mice [13], and the lack of a similar effect in DBA/2J mice.

For a number of reasons, it is unlikely that the lack of an effect of stress on reversal in the DBA/2J strain is due to insensitivity to the stress exposure itself. First, a number of studies have found that DBA/2J mice are typically more, not less, affected by stress than C57BL/6J mice, at least on measures of anxiety-like behavior [31]. Second, we confirmed that both strains showed robust activation of the hypothalamic-adrenal axis to stress, by assaying corticosterone 30 minutes after the third forced swim exposure (non-stressed C57BL/6J = 50.52±12.36 ng/mL, stressed C57BL/6J = 226.13±16.18 ng/mL, non-stressed DBA/2J = 66.09±12.96 ng/mL, stressed DBA/2J = 166.16±34.60 ng/mL). Serum corticosterone was measured from trunk blood collected after cervical dislocation and rapid decapitation, left to coagulate at room temperature for 1–2 hours, then centrifuged at 4°C for 30 seconds at 13,000 rpm and analyzed using an MP Biomedicals Radioimmunoassay for Corticosterone (bound and free), as previously described [60].

The most parsimonious explanation for the lack of a stress facilitation of reversal in DBA/2J mice may be that the excellent basal learning performance of this strain obscures any further improvement with stress (‘ceiling effect’). One implication of this finding is that future studies designed to test for facilitation of reversal learning by other manipulations may also be confounded by the basal learning phenotype in DBA/2J mice and should avoid this strain.

#### Re-reversal learning

It is unclear whether strain and stress differences in reversal learning are lost or are still evident when mice are required to learn a re-reversal. To test for this, beginning on the session after mice reached reversal criterion, mice were returned to the same stimulus-reward pairings learned on discrimination. There was a borderline significant strain×stress interaction for sessions (F1,41 = 4.07, *P* = .05), a main effect of strain on errors (F1,41 = 21.86, *P*<.01) and correction errors (F1,41 = 27.00, *P*<.01) to criterion (Figure S3A–C). There were significant main effects of strain on stimulus-reaction time (F1,41 = 38.30, *P*<.01) and reward-retrieval latency (F1,41 = 6.51, *P*<.05) (Figure S3D–E). Planned post hoc comparisons showed that stressed C57BL/6J took fewer sessions to complete re-reversal. Both stressed and non-stressed DBA/2J took fewer sessions, had fewer errors and correction errors, and had longer stimulus-response than stressed and non-stress C57BL/6J mice respectively. Non-stressed DBA/2J had shorter reward-retrieval latencies than non-stressed C57BL/6J.

During early re-reversal there was main effect of strain on sessions (F1,41 = 21.34, *P*<.01), errors (F1,41 = 22.56, *P*<.01), and correction errors (F1,41 = 24.86, *P*<.01) (Figure S3F–H) as well as stimulus-response (F1,41 = 31.71, *P*<.01) and reward-retrieval (F1,41 = 5.39, *P*<.05) (Figure S3I–J). Planned post hoc comparisons showed that stressed and non-stressed DBA/2J mice took fewer sessions, errors and corrections errors, and had longer stimulus reaction times relative to stressed and non-stressed C57BL/6J respectively. Non-stressed DBA/2J mice had shorter reward-retrieval latencies.

During late re-reversal there was a significant strain×stress interaction for sessions (F1,41 = 5.19, *P*<.05), a trend for an interaction for errors (F1,41 = 3.61, *P* = .06) and correction errors (F1,41 = 3.51, *P* = .07) (Figure S3F–H). There was a main effect of strain on stimulus-response (F1,41 = 36.71, *P*<.01) and reward-retrieval (F1,41 = 6.63, *P*<.05) (Figure S3I–J). Planned post hoc comparisons showed stressed C57BL/6J mice took significantly fewer sessions, errors and correction errors than non-stressed C57BL/6J mice. Non-stressed DBA/2J mice took fewer sessions, errors and corrections errors, and had longer stimulus-reaction times but shorter reward-retrieval latencies than non-stressed C57BL/6J mice.

These data make two main points. First, the faster learning phenotype in DBA/2J mice extends to re-reversal and, second, the ability of stress exposure to facilitate late stage reversal in C57BL/6J mice was still evident on re-reversal. This is especially remarkable considering mice had only been exposed to stress prior to reversal and demonstrates that this effect is persistent.

### BXD strains differ in touchscreen tasks

#### Pre-training

**Figure 4:**
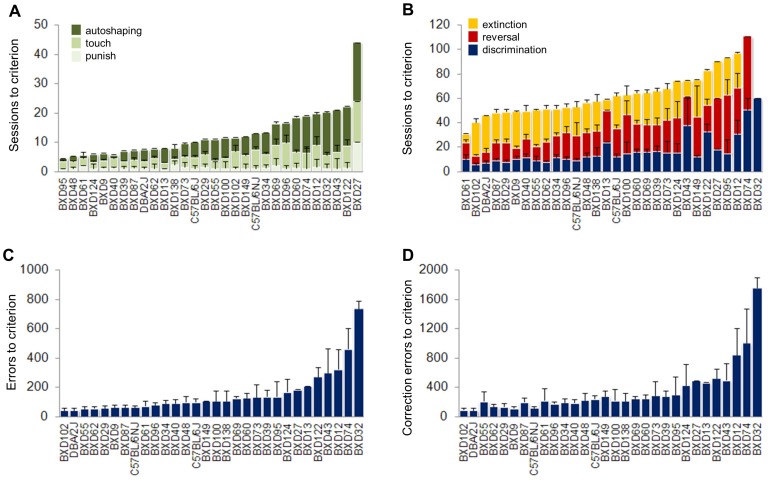
BXD-RI strain differences in training performance, and in discrimination and reversal learning. (**A**) Sessions to autoshaping, touch and punish criterion. (**B**) Sessions to discrimination, reversal and extinction criterion. Errors (**C**) and correction errors (**D**) to discrimination criterion. n = 1–16 per strain. Data are Means ± SEM.

There were significant strain differences in the number of sessions to attain criterion for the autoshaping (F29,168 = 5.75, *P*<.01), touch (F29,168 = 2.13, *P*<.01) and punish (F29,164 = 6.08, *P*<.01) stages of pre-training (**Figure 4A**). Strain differences were particularly marked for autoshaping, which simply required mice to collect pellets from the magazine within the allotted session duration. Difficulty with this task suggests some BXD strains may have required more experience with navigating the operant chamber and locating the pellets. Nonetheless, with the exception of BXD27, all strains completed pre-training and went on to testing for discrimination learning.

#### Discrimination

Strains differed significantly in sessions (F29,149 = 7.08, *P*<.01), errors (F29,149 = 10.44, *P*<.01) and correction errors (F29,129 = 10.29, *P*<.01) to discrimination criterion (**Figure 4B–D**), and also in average stimulus-reaction time (F29,149 = 2.27, *P*<.01) and reward-retrieval latency (F29,149 = 2.10, *P*<.01) (Figure S4A–B). BXD12, BXD32 and BXD74 were the most poorly performing strains, while BXD55, BXD102 and the DBA/2J parental strain performed best. The C57BL/6J parental strain ranked in the middle of the strains and was similar in learning to the C57BL/6NJ sub-strain.

Overall, these findings demonstrate marked and continuously distributed differences in discrimination learning across this 27-strain BXD panel. Major differences in discrimination learning were also reported in a 51-strain BXD panel that included 16 of the same strains currently tested [61]. The majority of the strains tested in both studies generally performed similarly. This did not, however, hold for all strains. For example, BXD102 learned relatively poorly in the Laughlin et al study [61], but was the best performer in the current analysis, while BXD12 ranked as a good learner in [61] and a poor learner in our study. These differences likely reflect differences in the tasks used, such as the greater spatial component of procedure of the task used by Laughlin et al.

#### Reversal

**Figure 5:**
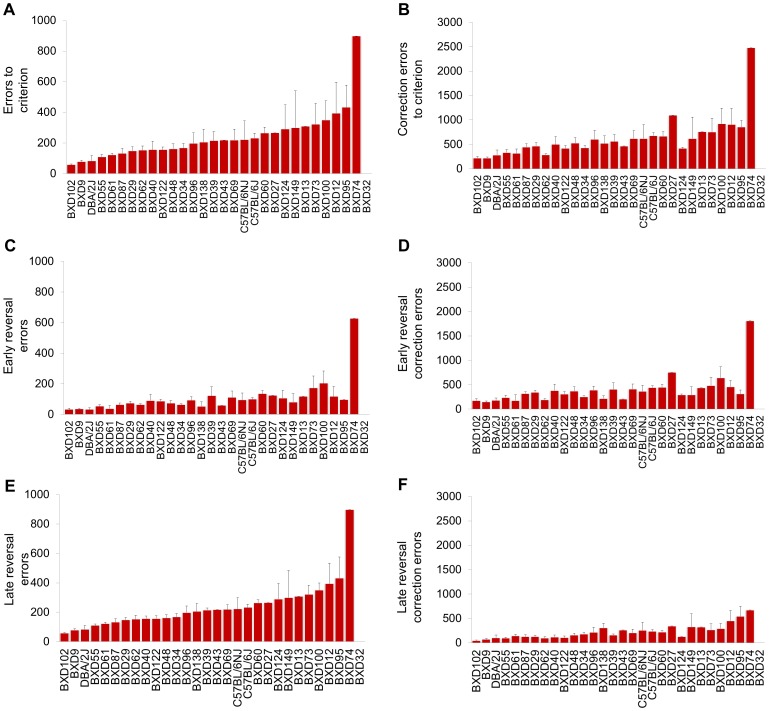
BXD-RI strain differences in reversal learning. Errors (**A**) and correction errors (**B**) to reversal criterion. Errors (**C**) and correction errors (**D**) made during early reversal. Errors (**E**) and correction errors (**F**) made during late reversal. n = 1–14 per strain. Data are Means ± SEM.

Strains differed significantly in sessions (F28,136 = 2.40, *P*<.01) (**Figure 4B**), errors (F28,136 = 2.44, *P*<.01) and correction errors (F28,136 = 3.04, *P*<.01) to discrimination criterion (**Figure 5A–B**), and in average stimulus-reaction time (F28,136 = 2.23, *P*<.01) (Figure S4C). Considering early reversal, strains differed significantly in errors (F28,136 = 3.06, *P*<.01) and correction errors (F28,136 = 3.33, *P*<.01) to reversal criterion, (**Figure 5C–D**). For the late reversal phase, strains differed significantly in errors (F28,136 = 2.19, *P*<.01) and correction errors (F28,136 = 2.23, *P*<.01) to reversal criterion (**Figure 5E–F**). The pattern of strain differences was roughly similar for the early and late reversal phases. It was also clear from this sub-phase breakdown that scores for reversal learning overall were largely driven by performance on late reversal.

The ranking of strain differences in reversal and discrimination learning were not equivalent. Some strains learned both tasks well (e.g., BXD102) or poorly (e.g., BXD74), but most differed to some extent, and in some cases (e.g., BXD95), strongly so. This lack of overlap was also reported in Laughlin et al.'s study [61] and suggests qualitative differences between discrimination and reversal learning. Another noteworthy similarity with this prior study was that, despite having callosal agenesis and cortical abnormalities, the BXD29 strain was not only unimpaired but was amongst the better performing strains on reversal. This is also generally consistent with the previous finding that prefrontal cortical lesions in C57BL/6J mice improve reversal learning in this task [13].

#### Extinction

As we had previously shown extinction differences between the C57BL/6J and DBA/2J parental strains in the touchscreen [15], we extended the analysis of the BXDs to include a measure of extinction. Strains did not differ in the number of sessions to extinction criterion (**Figure 4B**), but did differ significantly in errors (F27,126 = 2.54,*P*<.01) and correction errors (F27,126 = 1.87, *P*<.01) to criterion, and stimulus-reaction time (F27,126 = 1.96, *P*<.01) (Figure S5). The trait was continuously distributed, with BXD61, BXD13 and BXD43 being the best performing strains and DBA/2J, BXD29 and BXD124, the worst (see also Figure S5A,B). BXD61 was also amongst the good performers on the discrimination and reversal tasks, but overall the ranking of strains on extinction was not predicted by patterns in the other tasks, consistent with extinction as an independent measure of touchscreen behavior. This is borne out by the poor extinction in the DBA/2J strain, which contrasts with this strain's relatively superior discrimination and reversal learning. Given the earlier observation that DBA/2J mice also exhibit relatively deficient extinction of a simple touchscreen response to a single stimulus [15], this strain appears to have a pervasive extinction deficit across various touchscreen tasks.

#### Genetic correlations

Correlations between behavioral measures from the BXD-RI (and parental) strains are shown in **Table 1**. Different measures of learning (sessions to criterion, errors, correction errors) tended to correlate highly within a given learning task, whereas the same measures correlated less strongly between discrimination and reversal, and measures of extinction learning showed almost no correlation with the other tasks. These data indicate genetic independence of these three forms of learning. Given the selective effects of stress on late reversal reported above, the relatively modest genetic correlations between learning measures (particularly errors) on the early and late stages of reversal was also notable as evidence of genetic dissociability of the two stages. Other noteworthy findings to emerge from these data were the low genetic correlations between stimulus-response and reward-retrieval latencies with measures of learning.

**Table 1:** Genetic correlations between measures of touchscreen performance and learning.

		Pre	Discrimination					Reversal									Extinction			
		Pcr	Dcr	De	Dce	Dsr	Drr	Rcr	Re	Rce	ERe	ERce	LRe	LRce	Rsr	Rrr	Ecr	Ee	Ece	Esr
**Discrimination**	Dcr	0.28	-																	
	De	0.27	**0.92**	-																
	Dce	0.24	**0.82**	**0.89**	-															
	Dsr	0.21	0.36	0.20	0.20	-														
	Drr	0.04	0.11	0.08	0.05	0.23	-													
**Reversal**	Rcr	0.21	0.41	0.39	0.33	0.15	−0.01	-												
	Re	0.10	0.43	0.42	0.36	0.18	−0.03	**0.94**	**-**											
	Rce	0.15	0.23	0.22	0.14	0.02	0.03	**0.81**	**0.66**	-										
	ERe	0.26	0.40	0.41	0.33	0.15	0.01	**0.61**	**0.54**	**0.54**	-									
	ERce	0.16	0.33	0.33	0.26	0.06	−0.01	**0.75**	**0.63**	**0.84**	**0.74**	-								
	LRe	0.04	0.38	0.36	0.32	0.15	−0.04	**0.89**	**0.97**	**0.59**	0.32	0.50	-							
	LRce	0.02	0.28	0.31	0.28	−0.01	−0.02	**0.89**	**0.91**	**0.74**	0.45	**0.65**	**0.89**	-						
	Rsr	0.35	0.25	0.19	0.07	**0.54**	0.01	0.23	0.17	0.11	0.36	0.18	0.08	−0.01	-					
	Rrr	0.14	0.11	0.08	0.05	0.09	−0.01	0.18	0.12	0.10	0.40	0.21	0.02	0.04	0.28	-				
**Extinction**	Ecr	−0.10	−0.01	−0.05	0.01	0.05	−0.08	−0.04	−0.01	−0.01	−0.01	−0.06	−0.01	−0.02	0.01	0.01	-			
	Ee	−0.27	−0.05	−0.06	−0.05	−0.04	−0.10	−0.18	−0.12	−0.12	−0.13	−0.10	−0.11	−0.11	−0.05	−0.11	**0.74**	**-**		
	Ece	−0.23	−0.08	−0.10	−0.03	−0.01	−0.11	−0.17	−0.13	−0.11	−0.09	−0.09	−0.12	−0.10	−0.04	−0.09	**0.61**	**0.85**	**-**	
	Esr	0.02	0.10	−0.10	0.07	0.16	−0.03	0.15	0.19	0.10	0.05	0.09	0.20	0.12	0.21	0.03	**−0.39**	**−0.42**	**−0.39**	-

Correlations of >0.5 or <−0.5 are highlighted in larger underlined font. Pcr = sessions to pre-training criterion, Dcr = sessions to discrimination criterion, De = discrimination errors, Dce = discrimination correction errors, Dsr = discrimination stimulus-response latency, Drr = discrimination reward-retrieval latency, RCr = sessions to reversal criterion, Re = reversal errors, Rce = reversal correction errors, Ere = early stage reversal errors, ERcc = early stage correction reversal errors, LRe = late stage reversal errors, LRce = late stage reversal correction errors, Rsr = reversal stimulus-response latency, Rrr = reversal reward-retrieval latency, Ecr = sessions to extinction criterion, Ee = extinction errors, Ece = extinction correction errors, Esr = extinction stimulus-response latency.

### PCA structure of behavior

PCA of 15 behavioral measures taken during discrimination and reversal learning yielded a 5-factor structure accounting for 79% of the overall variance (**Table 2**). Factor 1 (accounting for 33% of the overall variance) was suggestive of a ‘reversal learning’ factor, and had the highest loading for sessions to reversal criterion, reversal errors and correction errors and early and late reversal correction errors. Factor 2 (16% of the overall variance) on the other hand, had only 2 loadings >3, late reversal errors and reward-retrieval latency on reversal trials. This indicates an association between a tendency to retrieve reward faster on reversal trials and a lower rate of error responding specifically during the late phase of reversal. To the extent that faster reward-retrieval latency demonstrates greater motivation, this factor could reflect ‘motivation-related late reversal learning.’

**Table 2:** Principal components analysis of touchscreen performance and learning.

	Factor 1	Factor 2	Factor 3	Factor 4	Factor 5
Sessions to pre-training criterion	0.19	0.06	0.19	**0.32**	**0.33**
Sessions to discrimination criterion	0.27	0.16	**0.36**	−0.11	−0.12
Discrimination errors	0.27	0.30	**0.39**	−0.20	−0.08
Discrimination correction errors	0.24	0.29	**0.41**	−0.24	−0.07
Discrimination stimulus-response latency	0.07	0.07	0.20	**0.56**	−0.13
Discrimination reward-retrieval latency	0.06	0.05	0.10	0.22	**0.81**
Sessions to reversal criterion	**0.35**	−0.21	−0.10	0.00	−0.07
Reversal errors	**0.41**	−0.13	−0.15	−0.05	0.07
Reversal correction errors	**0.39**	−0.23	−0.16	−0.02	0.00
Early stage reversal errors	0.23	0.28	**−0.35**	0.16	−0.19
Early stage correction reversal errors	**0.32**	−0.25	−0.11	0.07	−0.11
Late stage reversal errors	0.13	**0.47**	**−0.37**	0.02	0.04
Late stage reversal correction errors	**0.35**	−0.10	−0.13	−0.17	0.19
Reversal stimulus-response latency	0.09	−0.10	0.16	**0.59**	−0.31
Reversal reward-retrieval latency	−0.01	**0.54**	−0.29	0.13	0.00

Factor loadings >3 are highlighted in bold and larger font.

Factor 3 (14% of the overall variance) showed a clear contrast between discrimination (positive loadings >3 for sessions to discrimination criterion, discrimination errors and correction errors) and reversal learning (negative loadings >3 for early and late reversal errors). This suggests a ‘discrimination learning’ factor that associates closely with reversal performance, such that a high rate of errors on discrimination was associated with fewer errors on reversal (i.e., savings in reversal learning). Factor 4 (10% of the overall variance) had >5 loadings for stimulus-reaction time on discrimination and reversal, as well as a lower loading of sessions to pre-training criterion – indicating a task-independent ‘speed to respond’ factor. Factor 5 (7% of the overall variance) was another factor related to ‘motivation during discrimination,’ with a high loading for reward-retrieval latency on discrimination trials, and a lower loading for sessions to pre-training criterion.

Overall, this PCA analysis provides novel insight into the behavioral structure of learning and performance on these touchscreen-based tasks. The pattern of factor loadings suggested that discrimination and reversal learning are related but separable processes and that, even within the reversal task, the late stage of performance represents, to some extent, a distinct behavior. This latter finding speaks to the qualitatively distinct nature of late reversal learning, and is consistent with evidence in the current study and prior work that this stage of reversal is preferentially sensitive to manipulations such as stress and chronic ethanol exposure [13]–[14]. These statistical dissociations are also in line with the partial genetic independence of discrimination, reversal and late reversal learning found in our BXD-RI analysis. To extend these findings in future studies, it could be particularly valuable to examine a larger population of BXDs that enables well-powered testing for associations between these various measures of learning and specific genetic factors (e.g., via quantitative trait loci analysis). A final observation of note was that the highest loadings of measures of motivation (reward-retrieval latency) and speed to respond (stimulus-reaction latency) were weakly associated with measures of learning (with the exception of late reversal). This agrees with the low genetic correlations of these measures with indices of learning and lends further credence to the notion that these provide valuable ‘control’ measures of performance that are not confounded with learning.

## Conclusions

The main aim of the current study was to gain further insight into strain and genetic induced differences on learning in a mouse touchscreen-based pairwise visual discrimination and reversal learning paradigm. Results showed that these measures of discrimination and reversal learning are highly sensitive to strain differences, with some strains, including a number of albino strains, being severely impaired on even basic autoshaping and operant-shaping components of task performance, and other strains (e.g., C57BL/6J and DBA/2J) exhibiting robust learning-specific differences. The C57BL/6J and DBA/2J strains also showed varying responses to stress. While reversal learning was facilitated by stress in C57BL/6J mice, DBA/2J mice were unaffected, possibly because of a ‘ceiling effect’ in this strain's baseline performance that prevented further improvements caused by stress. Analysis of a panel of BXD-RI strains revealed marked variation across strains in discrimination and reversal learning indicative of genetically-complex and partially independent traits. Reinforcing these results, principal components analysis found that the main measures of discrimination and reversal learning loaded on independent factors. Together, these findings provide an important reference of strain differences in this behavioral platform, and should help inform the choice of appropriate strains and genetic backgrounds in future studies using these mouse touchscreen tasks to elucidate the neural and genetic basis of learning.

## Supporting Information

Figure S1 **Inbred strain differences in stimulus-reaction time and reward-retrieval latency.** Average stimulus-reaction time (**A**) and reward-retrieval latency (**B**) during discrimination. Average stimulus-reaction time (**C**) and reward-retrieval latency (**D**) during reversal. n = 9–27 per strain. Data are Means ± SEM.
(DOCX)

Figure S2 **Stress effects on stimulus-reaction time and reward-retrieval latency in DBA/2J and C57BL/6J mice.** Average stimulus-reaction time (**A**) and reward-retrieval latency (**B**) during discrimination. Average stimulus-reaction time (**C**) and reward-retrieval latency (**D**) during early and late reversal learning. n = 10–13 per stress group, per strain. †*P*<.05 non-stressed DBA/2J vs. non-stressed C57BL/6J, ‡*P*<.05 stressed DBA/2J vs. stressed C57BL/6J. Data are Means ± SEM.
(DOCX)

Figure S3 **Stress effects on re-reversal learning in DBA/2J and C57BL/6J mice.** Sessions (**A**), errors (**B**) and correction errors (**C**) to re-reversal criterion. Average stimulus reaction time (**D**) and reward latency (**E**) across re-reversal. Average stimulus reaction time (**D**) and reward latency (**E**) across re-reversal. Sessions (**F**), errors (**G**) and correction errors (**H**), and average stimulus reaction time (**I**) and reward latency (**J**) during early and late re-reversal. n = 10–13 per stress group, per strain. *P<.05 stressed vs. non-stressed control C57BL/6J, †*P*<.05 non-stressed DBA/2J vs. non-stressed C57BL/6J, ‡*P*<.05 stressed DBA/2J vs. stressed C57BL/6J. Data are Means ±SEM.
(DOCX)

Figure S4 **BXD-RI strain differences in stimulus-reaction time and reward-retrieval latency.** Average stimulus-reaction time (**A**) and reward-retrieval latency (**B**) during discrimination. Average stimulus-reaction time (**C**) and reward-retrieval latency (**D**) during reversal. n = 1–14 per strain. Data are Means ± SEM.
(DOCX)

Figure S5 **BXD-RI strain differences in extinction learning.** Errors (**A**), correction errors (**B**) to extinction criterion and average stimulus-reaction times (**C**). n = 1–14 per strain. Data are Means ± SEM.
(DOCX)
