# Supplementary figures and images for: Strains and Stressors: An Analysis of Touchscreen Learning in Genetically Diverse Mouse Strains
Source: PLoS One. 2014 Feb 19;9(2):e87745. doi: 10.1371/journal.pone.0087745 (PMC3929556; doi:10.1371/journal.pone.0087745)

**Figure S1**


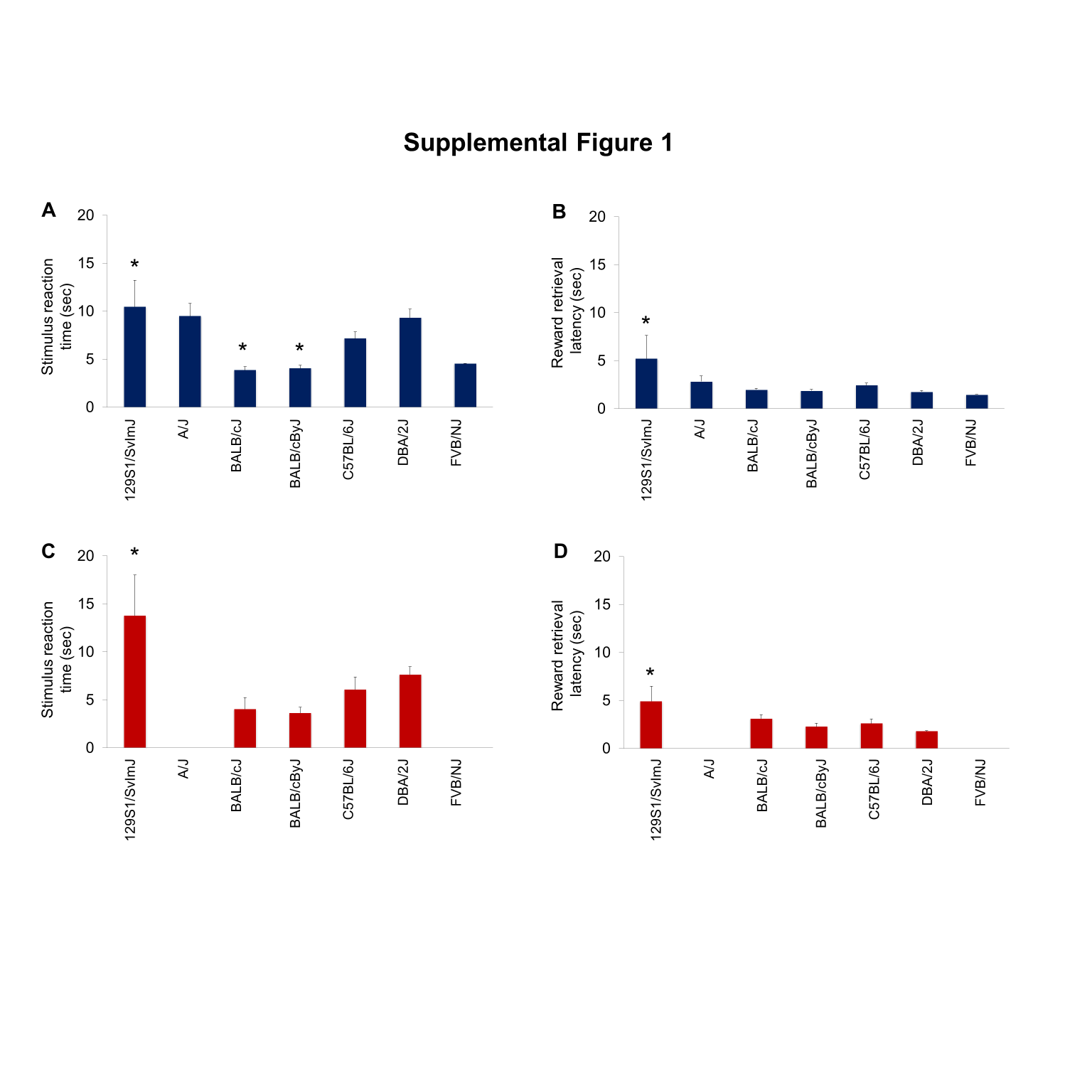

Supplement: Figure S1 — Inbred strain differences in stimulus-reaction time and reward-retrieval latency. Average stimulus-reaction time (A) and reward-retrieval latency (B) during discrimination. Average stimulus-reaction time (C) and reward-retrieval latency (D) during reversal. n = 9–27 per strain. Data are Means ± SEM. (DOCX) [file pone.0087745.s001.docx]

**Figure S2**


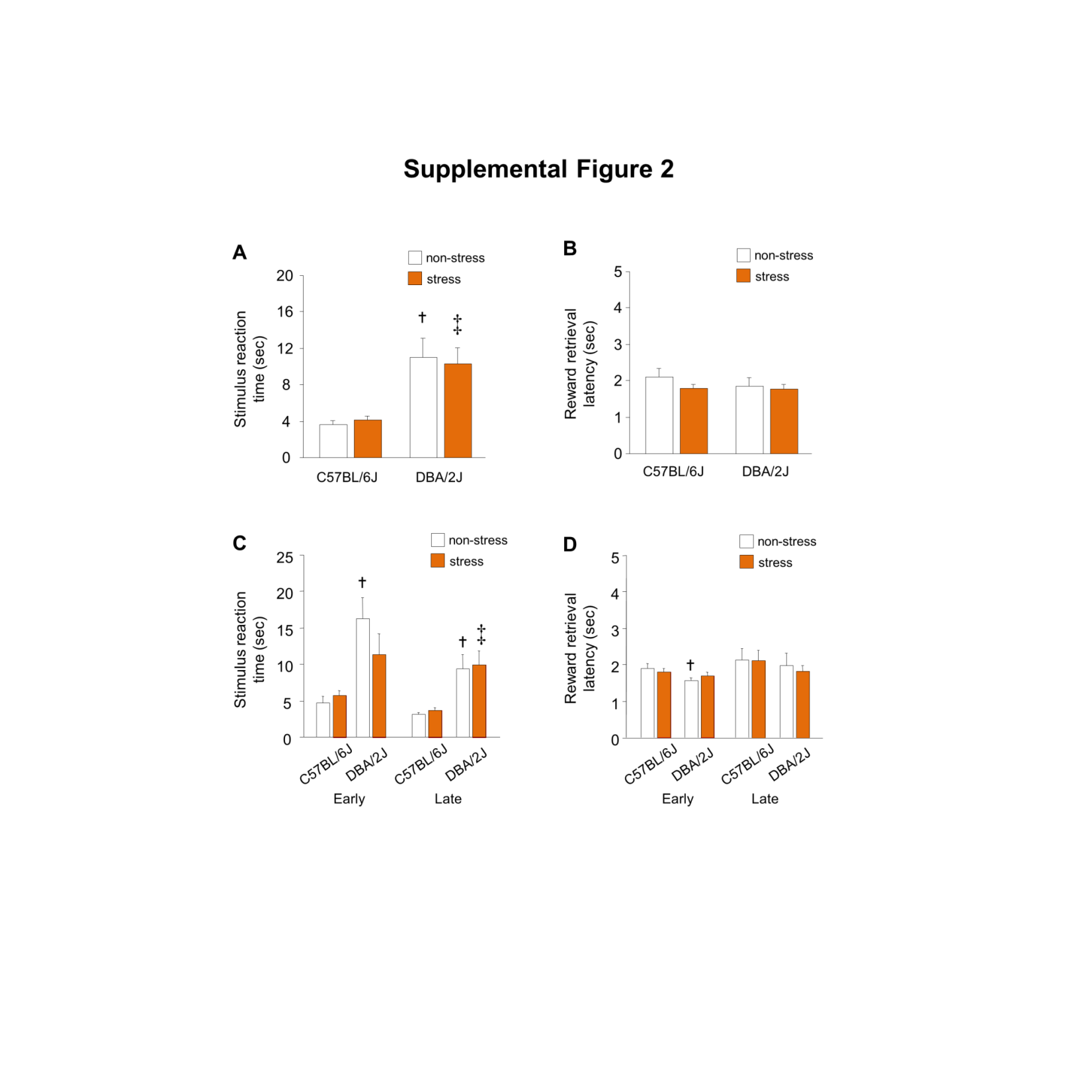

Supplement: Figure S2 — Stress effects on stimulus-reaction time and reward-retrieval latency in DBA/2J and C57BL/6J mice. Average stimulus-reaction time (A) and reward-retrieval latency (B) during discrimination. Average stimulus-reaction time (C) and reward-retrieval latency (D) during early and late reversal learning. n = 10–13 per stress group, per strain. †P<.05 non-stressed DBA/2J vs. non-stressed C57BL/6J, ‡P<.05 stressed DBA/2J vs. stressed C57BL/6J. Data are Means ± SEM. (DOCX) [file pone.0087745.s002.docx]

**Figure S3**


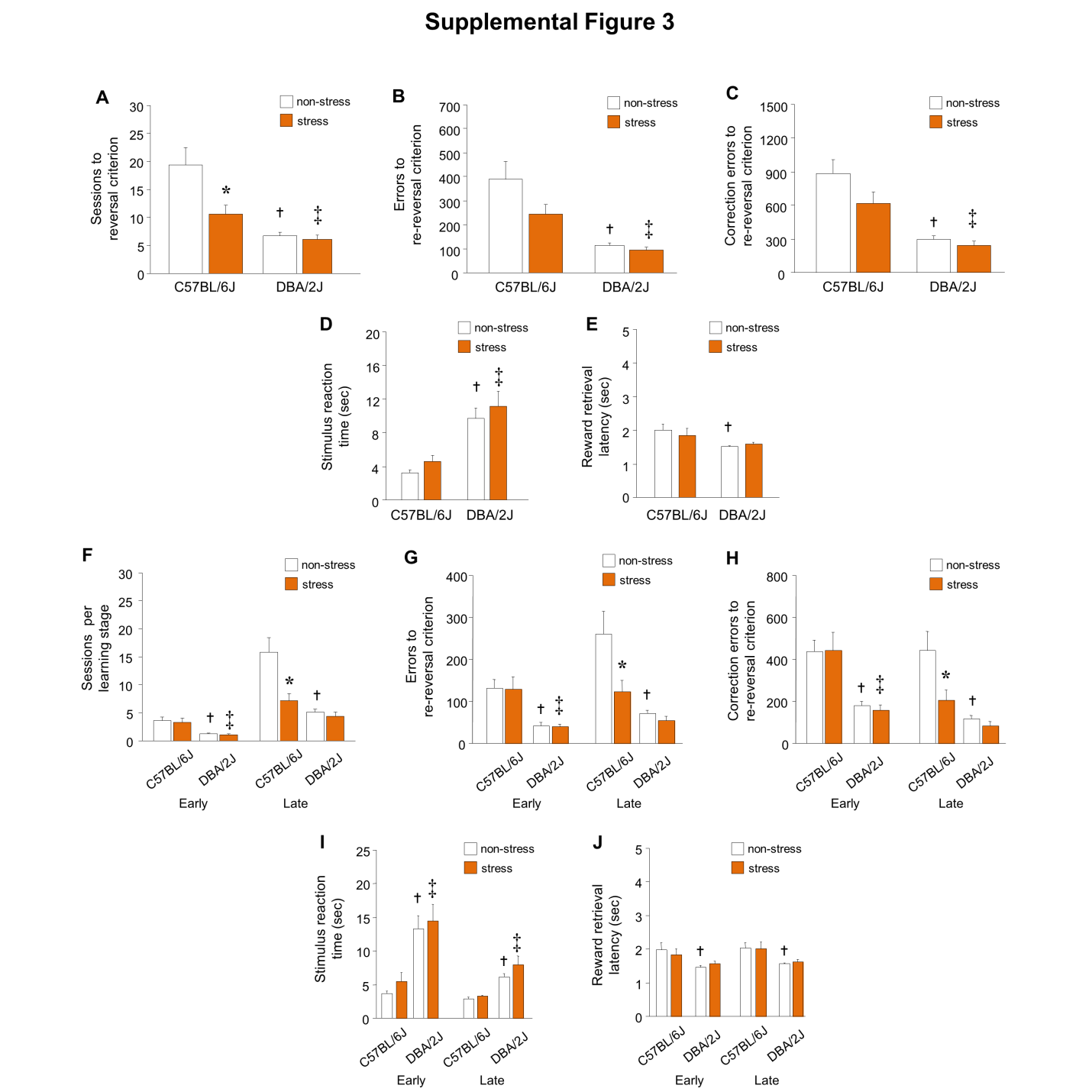

Supplement: Figure S3 — Stress effects on re-reversal learning in DBA/2J and C57BL/6J mice. Sessions (A), errors (B) and correction errors (C) to re-reversal criterion. Average stimulus reaction time (D) and reward latency (E) across re-reversal. Average stimulus reaction time (D) and reward latency (E) across re-reversal. Sessions (F), errors (G) and correction errors (H), and average stimulus reaction time (I) and reward latency (J) during early and late re-reversal. n = 10–13 per stress group, per strain. *P<.05 stressed vs. non-stressed control C57BL/6J, †P<.05 non-stressed DBA/2J vs. non-stressed C57BL/6J, ‡P<.05 stressed DBA/2J vs. stressed C57BL/6J. Data are Means ±SEM. (DOCX) [file pone.0087745.s003.docx]

**Figure S4**


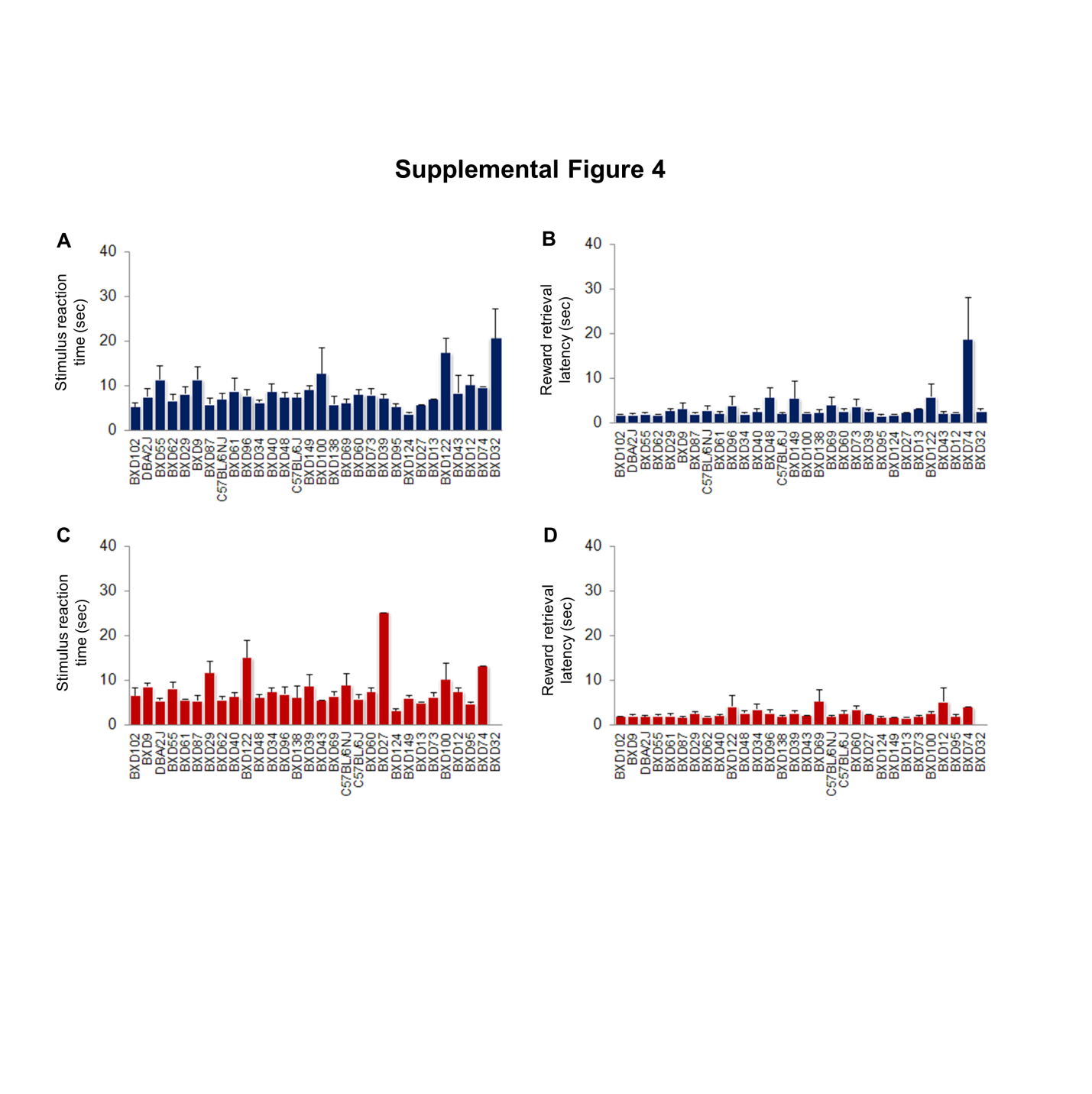

Supplement: Figure S4 — BXD-RI strain differences in stimulus-reaction time and reward-retrieval latency. Average stimulus-reaction time (A) and reward-retrieval latency (B) during discrimination. Average stimulus-reaction time (C) and reward-retrieval latency (D) during reversal. n = 1–14 per strain. Data are Means ± SEM. (DOCX) [file pone.0087745.s004.docx]

**Figure S5**


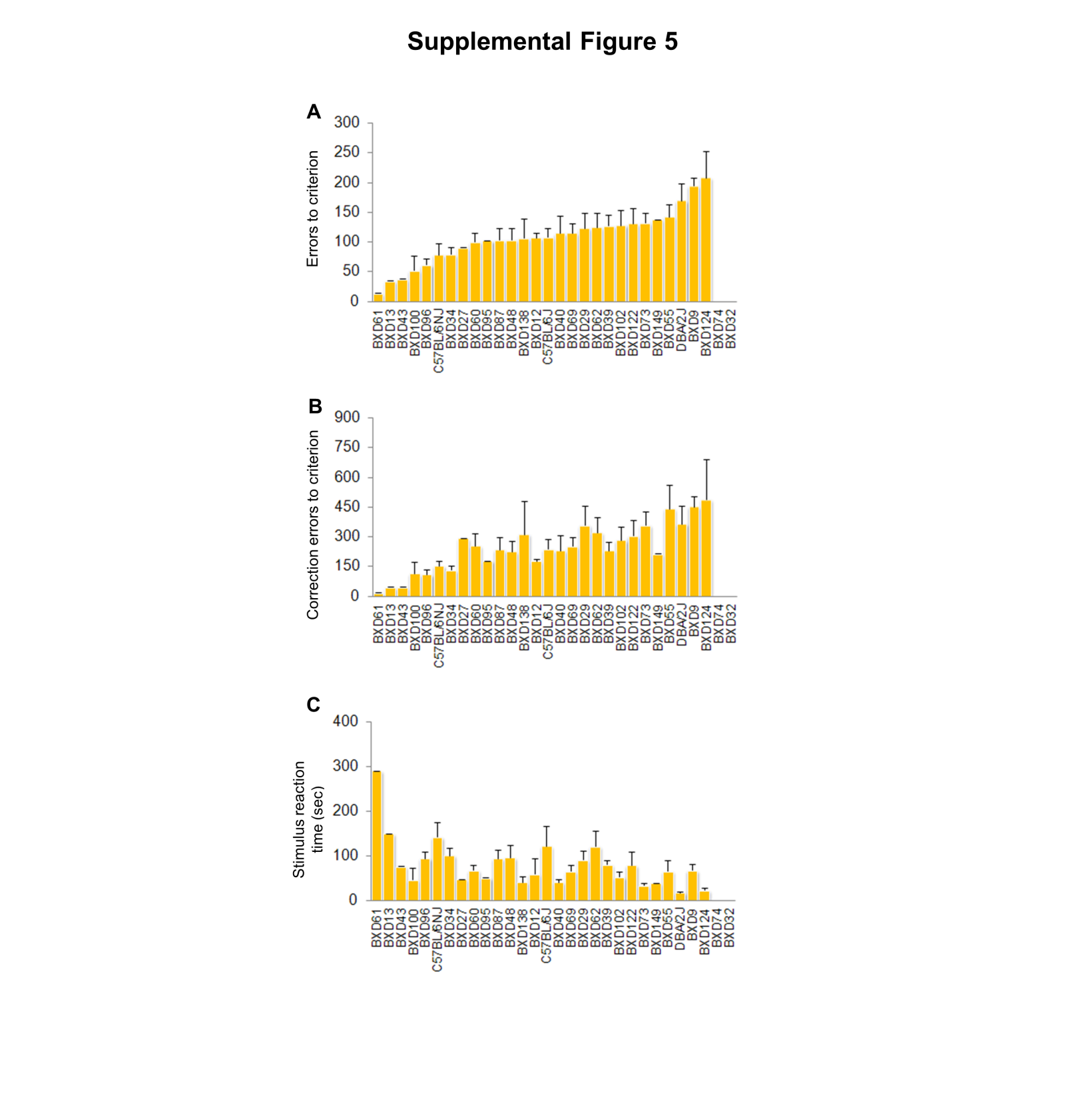

Supplement: Figure S5 — BXD-RI strain differences in extinction learning. Errors (A), correction errors (B) to extinction criterion and average stimulus-reaction times (C). n = 1–14 per strain. Data are Means ± SEM. (DOCX) [file pone.0087745.s005.docx]
